# Hunting dog behaviour is a key driver impacting harvest quantity and quality of truffles

**DOI:** 10.1038/s41598-025-93116-z

**Published:** 2025-03-13

**Authors:** Paul W. Thomas, David Kothamasi

**Affiliations:** 1 Faculty of Natural Sciences, University of Stirling, Stirling, FK9 4LA UK; 2 Mycorrhizal Systems Ltd, Lancashire, PR25 2SD UK; 3 Laboratory of Soil Biology and Microbial Ecology, Department of Environmental Studies, University of Delhi, Delhi, 110 007 India

**Keywords:** Truffle, Ecophysiology, Socioeconomic, Scent dog behaviour, Truffle harvesting, *Tuber aestivum*, Dog handler, Ecophysiology, Behavioural ecology, Ecosystem ecology

## Abstract

Truffles are an iconic food that have long held high regard. Here we explore the seasonality and eco-physiological interactions affecting truffle quality and quantity across time and space. Collaborating with professional truffle hunters working eight different locations, detailed metrics of 3180 recovered truffles from 236 hunt events and spanning a full fruiting period, were recorded. Contrary to expectations, truffle weight showed no correlation with climate variables, suggesting a limited influence of environmental factors such as temperature and precipitation on truffle size. We also found that truffle maturity and damage from mycophagy were strongly linked, with deeper truffles being more mature but also more susceptible to damage. Finally, we observe that scent-dog behaviour significantly impacts the quantity and quality of recovered truffles, and we address the necessity of considering this in truffle ecophysiology studies. Alongside advances in our biological understanding, we make recommendations of how training methods can be improved to lead to greater detection and quality targeting with immediate socioeconomic impact. These findings highlight the complex interplay between truffle physiology, environmental factors, and human and animal behaviours, emphasizing the need for further considered research to enhance our understanding of truffle biology and to improve truffle cultivation practices.

## Introduction

Truffles are fruiting bodies of the Ascomycetous genus *Tuber* that form ectomycorrhizal symbioses with gymnosperms and angiosperms. They have spawned a global industry with harvesting activities spanning six continents^1,2^. Originating in Europe, the economic impact within just Italy is estimated to be more than €100 million with ~ 180,000 truffle hunters/cultivators dedicated to the field^2^. As a hypogeous fruiting body that occurs in low density, harvesting has alluded mechanisation and collection is largely dependent on trained scent detection dogs^3^ leaving much room for optimisation.

Despite the economic interest, there are many facets of truffle ecology and biology that remain to be elucidated. As a hypogeal fruiting body that lacks spore ejective tissues, truffles have a dependence on mycophagy for ascospore dispersal and several vertebrate and invertebrate species have been shown to act as vectors^4–6^. However, basic questions such as how mycophagy relates to truffle fruiting depth, have not previously been addressed. The assembly of large truffle-collection records is one approach that allows us to explore truffle ecophysiology at scale, and this has been used by previous authors such as Büntgen et al*.*^7^*,* to describe a lack of relationship between weight and maturity for *T. aestivum*. However, many other relationships remain to be explored. Moreover, investigations based on fruitbody collection are necessarily dependant on scent detection dogs and the behaviours or ethology of such are often not acknowledged in the interpretation of previous studies. Given that ~ 42% of all fruitbodies may remain undetected^8^, we posit that truffle-dog and handler ability must be considered in the interpretation of data from such studies. Here, we aim to acknowledge such variables in interpretation whilst using a comprehensive approach spanning eight different hunt locations and 236 hunt events, to explore truffle ecophysiology in greater depth than has previously been attempted. Additionally, collecting data across a whole harvesting season, spanning five months, will allow us to address how parameters such as truffle weight and maturity are impacted by seasonal progression. This detailed and overarching approach will allow a complex analysis of truffle ecophysiology across a broad spatiotemporal scale whilst acknowledging how truffle dog ethology and human interaction may be influential in the collected data. The results will be of benefit not only for those interested in truffle ecophysiology but also of immediate importance and relevance to practitioners.

## Materials and methods

### Study design

Most truffle ecophysiology studies depend on one site or a small number of collections^9^. Elevating this study required a significant increase in replicate numbers and therefore collaboration with professional truffle hunters was needed. We recruited a professional truffle hunting group in central Greece, who were subsequently trained for systematic study and in measuring different truffle parameters. Aside from data collection, the hunters were instructed to continue with hunt activity as they would in a normal harvesting season. This passive approach allowed the hunt activity to remain uninfluenced nor directed by us and resulted in 236 separate hunt events over a five-month period. Towards the end of the harvest window the season ended abruptly, with a subsequent visit one week after the end of the study resulting in only two small truffles found across all eight sites. This abrupt seasonal end is common in this region of Greece and the subsequent visit confirms this as accurate.

This passive study included no experimentation on either animal or humans. Truffle hunters were asked to continue their activities as normal with no direction, interference or interaction aside from a request to provide recorded details. This study is solely focussed on database interpretation from ‘normal’ activities and does not include experimentation involving animals nor humans. Truffle hunters provided full consent and willingly provided harvesting logs to the study, the need for informed consent and approval has been waived. The University of Stirling’s General University Ethics Panel which undertakes review of human participant research classified the work as analysis of a secondary dataset and stated that the project would not require further approval. All methods were performed in accordance with the relevant guidelines and regulations.

### Area and in-field fruiting body locating

Eight separate natural truffle hunting areas producing *Tuber aestivum* Vittad. were selected on the grounds that they were well known to the truffle hunters, were within close enough proximity to allow frequent visitation and it was understood that no other hunters visit these sites. All sites were located within central Greece and were dominated by *Quercus coccifera*, *Quercus pubescens*, and *Carpinus betulus* trees aged 10–35 years. All sites were located within a circumferential radius of 107.7 km, had an altitude variance of 154 m (117 m − 271 m) and all data was collected in 2023. Pertinent to truffle mycophagy, the area has several rodent species (vertebrates) as well as invertebrates such as isopods and gastropods^4–6^.

The hunting team included three full-time commercial harvesters with ~ 20 years’ experience, working a rotating team of seven trained truffle scent detection dogs. The dogs were largely crossbreeds between Pointer, Springer Spaniel and Labrador although one was a pure bred Lagotto Romagnolo. On each hunt event multiple dogs were employed, and the air temperature was logged. Air temperatures rather than soil temperatures were logged because a key objective of this study was to allow the hunters to employ methods they would use in a routine hunting season. Moreover, the effects of soil temperature as well as soil physical and chemical characteristics on truffle physiology have been extensively documented by other workers^10–12^. Each time a truffle was located, the depth of the fruiting body was also recorded. Depth here represents the distance from the soil surface to the top of the truffle itself.

### Post-harvest fruiting body analysis

All collected fruiting bodies were stored at ~ 4 °C for a maximum of 12 h prior to analysis. Samples were removed from their collection bags and brushed free from adhering matter. Each sample was weighed and then assessed on a 3-point scale for the degree of mycophagy damage. The categories used were: 1- free from damage, 2- evidence of mycophagy with ~ 1 − 30% of the surface area impacted, and 3- high degree of mycophagy with + 30% of the surface area impacted. The maturity of each sample was also recorded using a 5-point scale based on colour variation of the gleba^7^.

### Statistical analyses

Statistical analyses were implemented on R version 4.3.2^13^. Data were tested for Gaussian distribution using the Shapiro–Wilk test. As the Shapiro–Wilk test in R is limited to data with sample size ≤ 5000, when sample size was > 5000, we employed the Anderson–Darling test for normality using the R-package *nortest* version 1.0–4^14^. Data that did not follow the Gaussian distribution were transformed with R-package *bestNormalize* version 1.9.0^15^ using the function bestNormalize. Differences in the means were evaluated with a one-way analysis of variance (ANOVA). Pair-wise differences were tested post-hoc with Tukey’s honest significant differences. Relationships between the parameters studied were evaluated using Pearson’s correlation. Average values of truffle weight, maturity and damage grades, depth of collection, and temperature calculated for each hunting day were used to implement correlations with total truffle number collected each hunting day. Relationships between truffle weight, maturity and damage grades, depth and temperature were evaluated by performing correlation analyses using all 3180 observations recorded from the eight sites between March and July. Prior to performing the correlations all the values were scaled using the scale function of R. Correlation matrices were constructed using the R-packages *corrplot* and *ggcorrplot* versions 0.92 and 0.1.4.1 respectively^16,17^. A partial least squares structural equation model (PLS-SEM) was constructed to ascertain the causal factors that guided dog ethology and truffle hunters. The PLS-SEM was built using the R-package SEMinR version 2.3.2^18^. Location, ambient site temperature, depth of truffles, quality (maturity, damage and weight of truffles), numbers of truffles harvested, and hunt interval were included as constructs in the PLS-SEM. A bootstrapping method with 1000 replications was implemented to evaluate significance of the path coefficient values (*R*^2^) of the causal relationships between the tested variables.

## Results

**Fig. 1:**
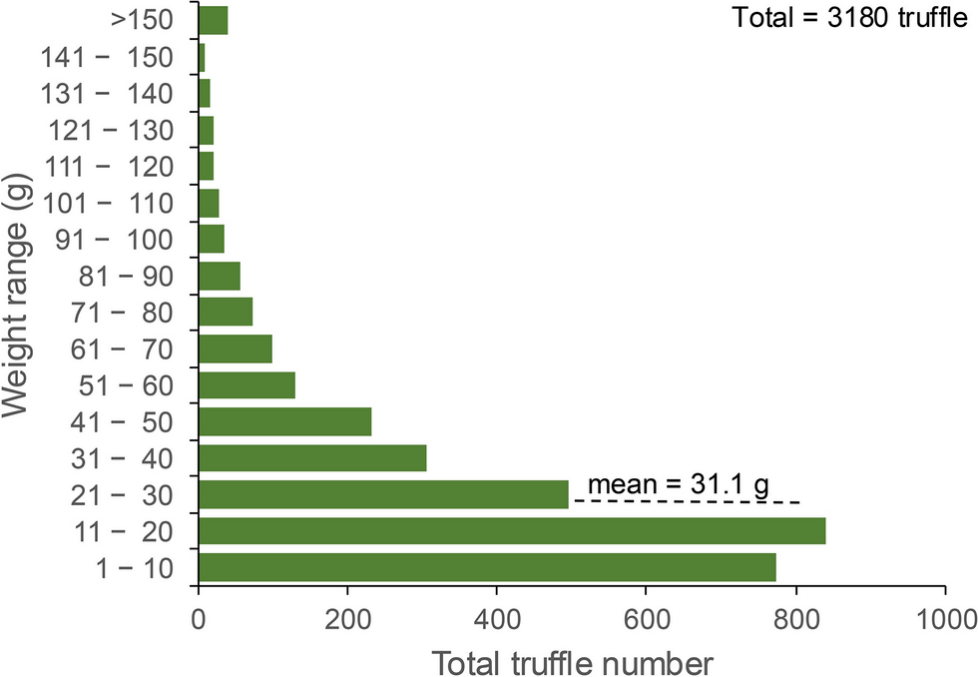
Weight distribution range in the 3180 T*. aestivum* truffles collected from eight sites over a five-month harvesting period from March to July.

**Fig. 2:**
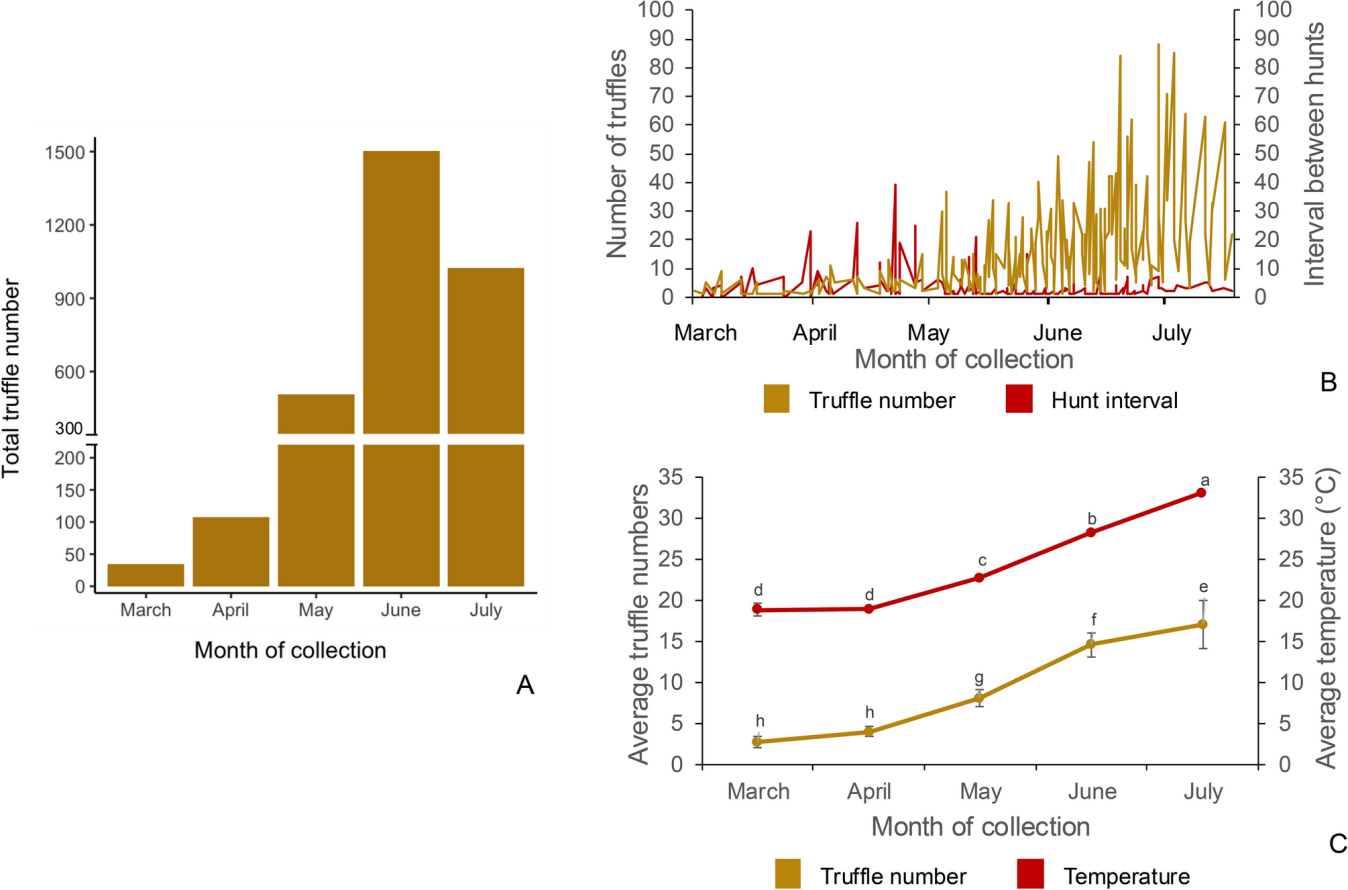
Truffle hunt. (**A**) number of truffles collected in each month; (**B**) intervals between truffle hunting events in each month; (**C**) average monthly temperature and average numbers of truffles collected each month. Error bars represent standard error of the mean. Values that do not share a letter are significantly different (*p* ≤ 0.05) in a Tukey’s post-hoc test for pairwise differences.

**Fig. 3:**
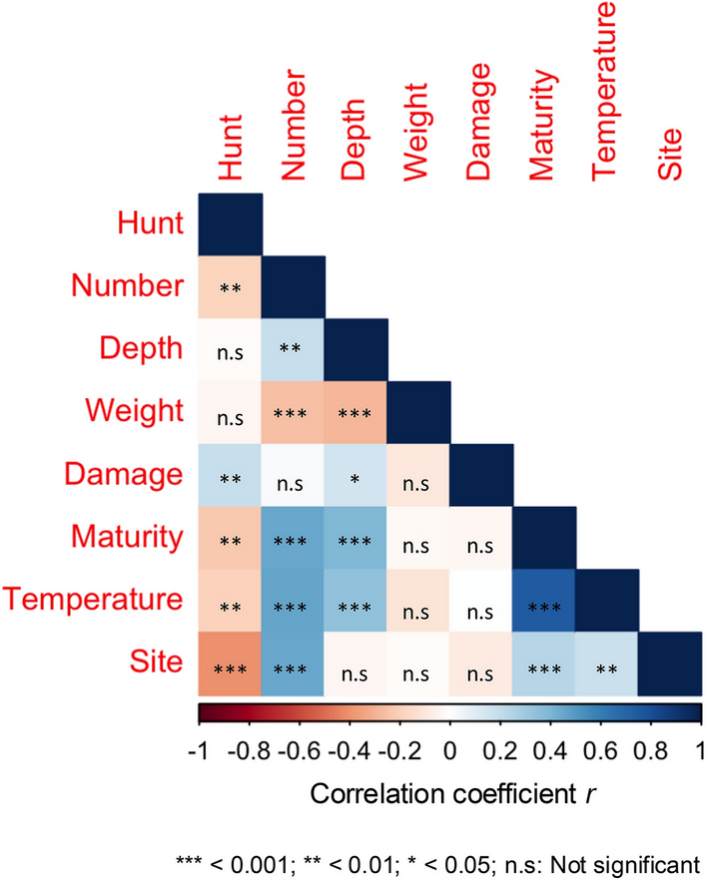
Correlation matrix showing the interrelationships between site, hunt intervals, truffle numbers, truffle quality characteristics, depth of collection and temperature. Average values of weight, maturity and damage grades, depth and temperature were used to determine the correlations.

**Fig. 4:**
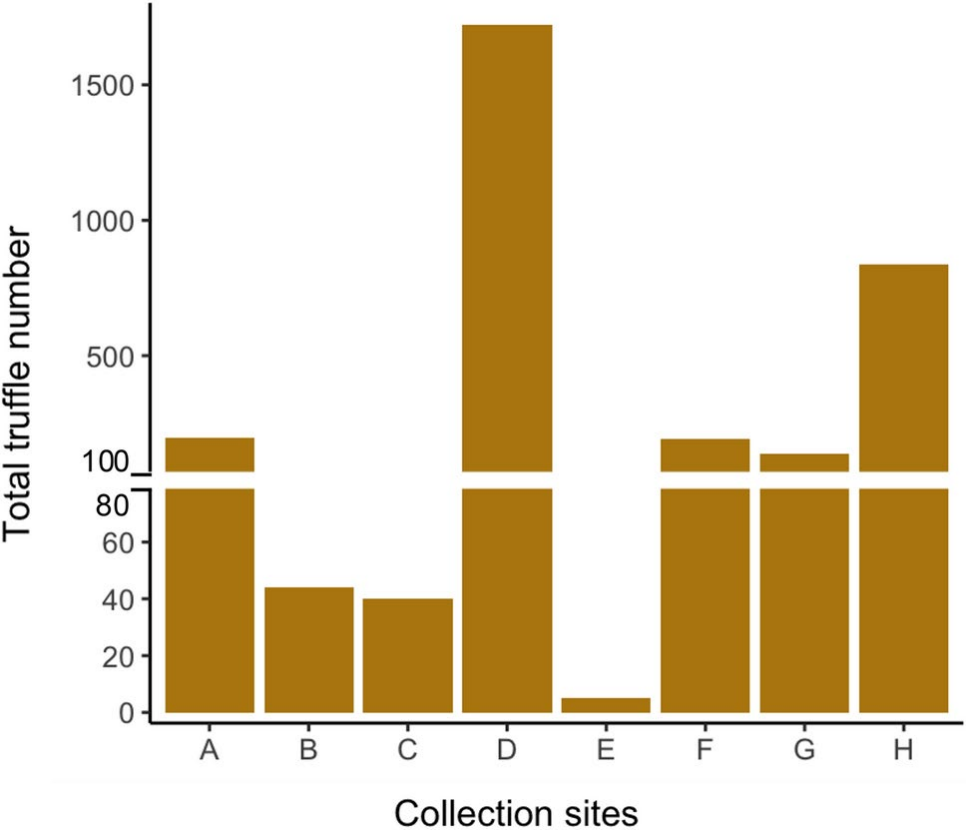
Total number of *T. aestivum* truffles collected from each site over a five-month harvesting period from March to July.

The hunters invested 236 days in harvesting 3180 truffles from eight sites over a period of five months spanning March to July. These truffles weighed between 1 – 378 g with an average weight of 31.1 g (Fig. 1; Supplementary Fig. 1A, B). Month and site of collection impacted the truffle quality and efficiency of hunt. The highest numbers of truffles (1504) were collected in June while the lowest numbers were collected in March (Fig. 2A). Periods of high harvest overlapped with numbers of days spent on the hunt (Fig. 2B). In other words, harvest quantity correlated negatively with the length of interval between hunts (*r* =  − 0.19, *p* = 0.004; Fig. 3). The average truffle numbers ranged from 2.6 in March to 16.9 in July, during the same period the average temperatures were 18.8 °C and 33.1 °C respectively (Fig. 2C). Site D was the most productive among the eight sites with a total harvest of 1724 truffles. In contrast, with only five truffles harvested, site E provided the lowest yield (Fig. 4). However, there was a large site-dependent variance in the numbers of truffles harvested.

**Fig. 5:**
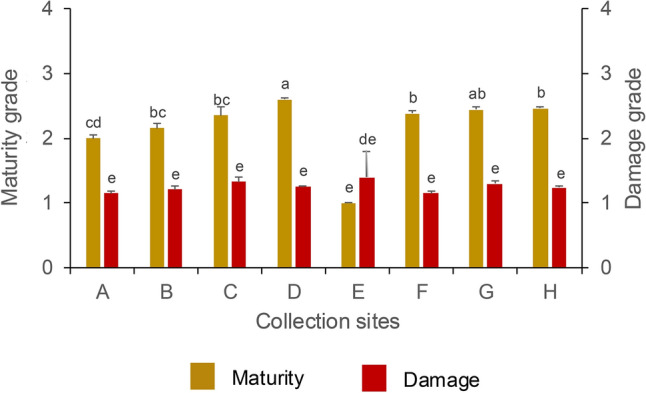
Average maturity and damage grades of *T. aestivum* truffles harvested at each collection site. The error bars represent standard error of the mean. Values that do not share a letter are significantly different (*p* ≤ 0.05) in Tukey’s post-hoc test for pairwise differences.

Average damage grade was < 2 in all the sites and differences between the sites were not significant (Fig. 5). We did not find any correlation between site and damage grade (Fig. 3). Average maturity grades across sites A – H were between 2 – 3 and sites D and G had the truffles with highest maturity grades (Fig. 5). Maturity grade correlated positively with site of truffle collection (*r* = 0.25, *p* = 0.0001; Fig. 3).

Truffles of maturity grades 3 and 4, respectively had lower average weights than truffles of maturity grades 1 and 2 (*F*_3,3176_ = 18.1, *p* < 0.0001; Supplementary Fig. 1A). Truffles of maturity grade 4 had the highest damage grade but no differences in damage grade were found in truffles of maturity grades 2 – 3 (Supplementary Fig. 1B). While truffle weight correlated negatively (*r* =  − 0.02, *p* < 0.0001) with maturity grade, we did not find a significant relationship between weight and damage grade (Fig. 3).

**Fig. 6:**
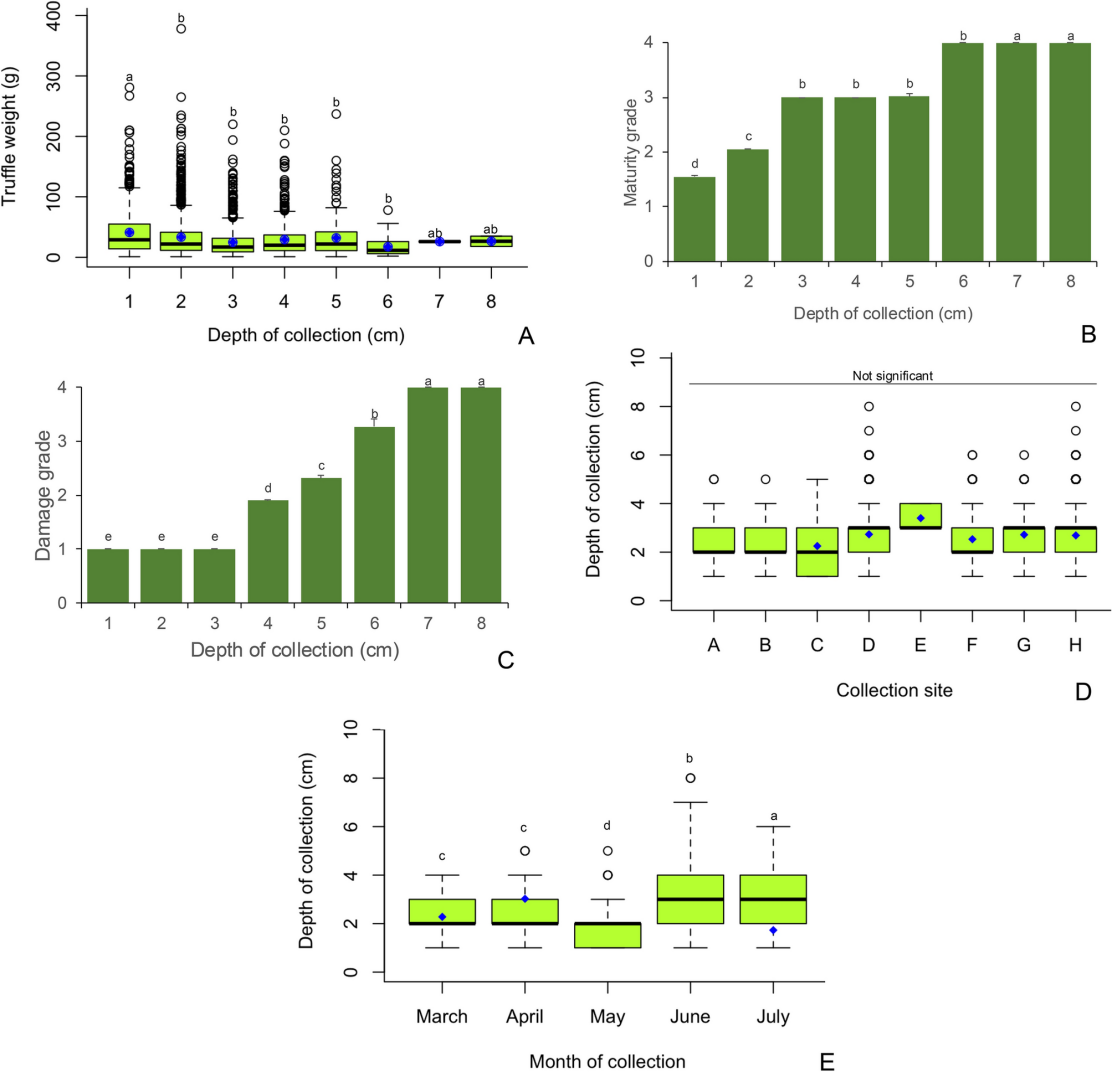
Impact of collection depth on (**A**) truffle weight; (**B**) truffle maturity grade; (**C**) truffle damage grade; and the influence of (**D**) collection site and (**E**) month of collection on the depth of truffle harvest. Values that do not share a letter are significantly different (*p* ≤ 0.05) in Tukey’s post-hoc test for pairwise differences. The centre line in the box and whisker plots indicates the median and the blue dots denote the mean. The upper and lower whiskers indicate the highest and lowest values while circles above whiskers are the outliers. Error bars in the bar charts represent standard error of the mean.

Even though the average truffle weights were not correlated with depth of collection (Fig. 3), when individual weights of the 3180 harvested truffles were compared with the depth of collection, a significant negative relationship was observed (*r* =  − 0.11, *p* < 0.0001; Supplementary Fig. 2). Average weights were highest for truffles collected from a depth of 1 cm (*F*_7,3171_ = 14.58, *p* < 0.0001). No significant differences were found in average weights for truffles collected at depths of 2 – 6 cm. Moreover, the truffles collected from 7 and 8 cm depths did not differ in average weights from those collected at 1 cm depth (Fig. 6A). Only two truffles each were collected from depths of 7 and 8 cm.

**Fig. 7:**
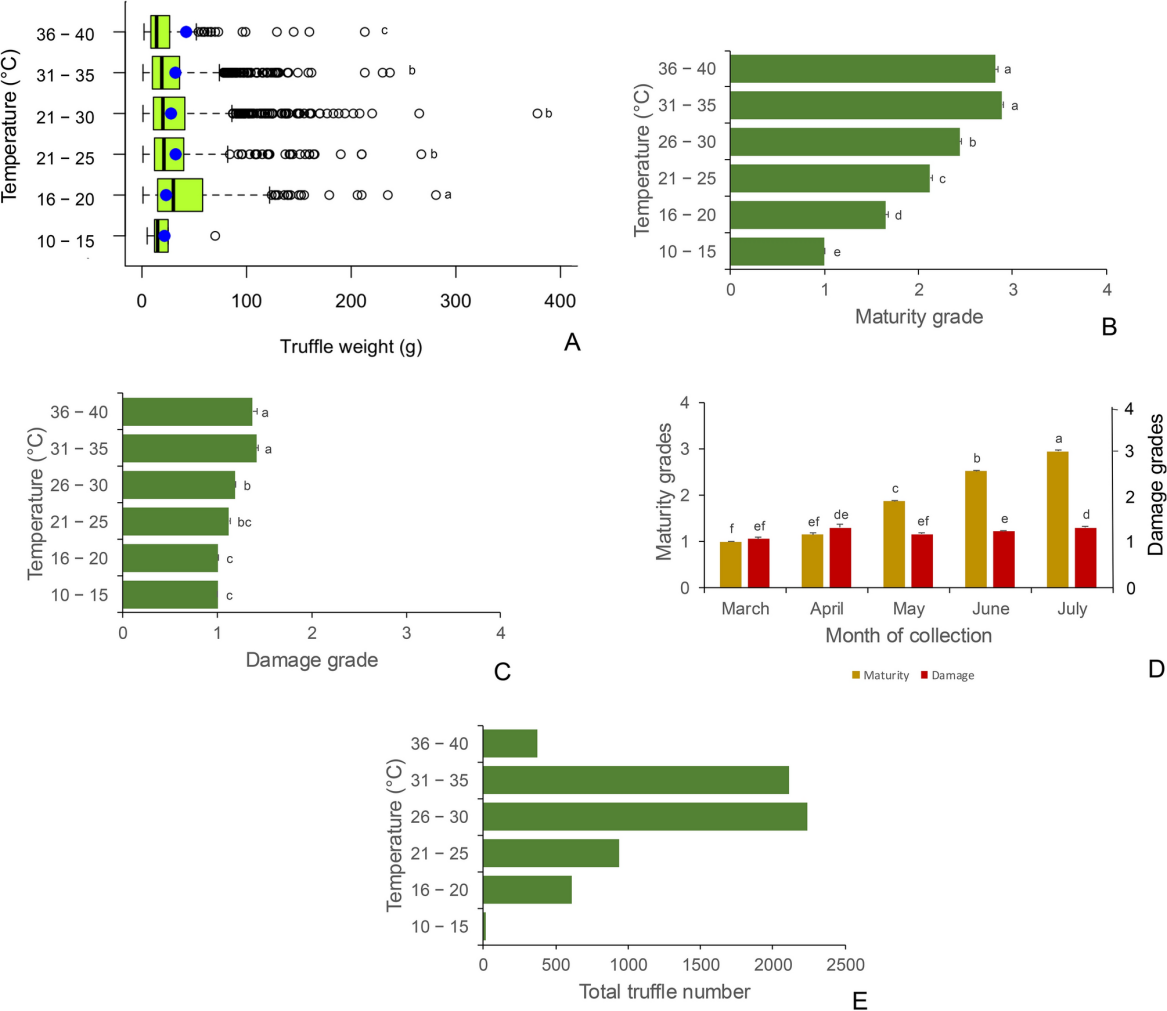
Effects of temperature on (**A**) truffle weight; (**B**) maturity grade; (**C**) damage grade; (**D**) month-wise impact on maturity and damage grades; and (**E**) total truffle number. Values that do not share a letter are significantly different (*p* ≤ 0.05) in Tukey’s post-hoc test for pairwise differences. The centre line in the box and whisker plot indicates the median and the blue dots denote the mean. The left and right whiskers indicate the lowest and highest values while circles to right of the whiskers are the outliers. Error bars in the bar charts represent standard error of the mean.

Depth of collection impacted the truffle quality. Maturity (*r* = 0.40, *p* < 0.0001) and damage grades (*r* = 0.15, *p* = 0.01) of the truffles had a significant positive correlation with depth of collection (Fig. 3). Maturity (*F*_7,3171_ = 4470, *p* < 0.0001) and damage grades (*F*_7,3171_ = 1535, *p* < 0.0001) were highest at depths of 6 – 8 cm and lowest at depths of 1 – 2 cm (Fig. 6B, C). There were no site-based differences in the depths at which the truffles were hunted in the eight collection sites (Fig. 6D). However, the hunters were digging deeper for truffles in the months of June and July, compared to March and April (*F*_4,3171_ = 178, *p* < 0.0001; Fig. 6E). Indeed, depth of collection correlated positively with temperature (*r* = 0.35, *p* < 0.0001; Fig. 3) which was higher in the months of June and July compared to March and April (*F*_4,3138_ = 1393, *p* < 0.0001). Truffle quality indicators such as maturity (*r* = 0.76, *p* < 0.0001), damage (*r* = 0.09, *p* < 0.0001) and weight (*r* =  − 0.14, *p* < 0.0001) correlated significantly with temperature (Supplementary Fig. 2). The average truffle weight was highest in the temperature range of 16 – 20 °C and lowest at temperatures of 36 – 40 °C (Fig. 7A). Truffle maturity and damage grades were highest in temperature ranges of 31 – 40 °C (Fig. 7B, C). Truffle maturity and damage were lowest in the cooler temperature regimen of 10 – 20 °C. These temperatures coincided with March and April. While truffles with average maturity grades of 3 – 4 were harvested in June and July, there were no differences in the damage grades of the truffles in any of the months (Fig. 7D). Truffle harvest was highest in the temperature ranges of 26 – 35 °C (Fig. 7E). These were the temperature ranges in June and July (Fig. 2C).

**Fig. 8:**
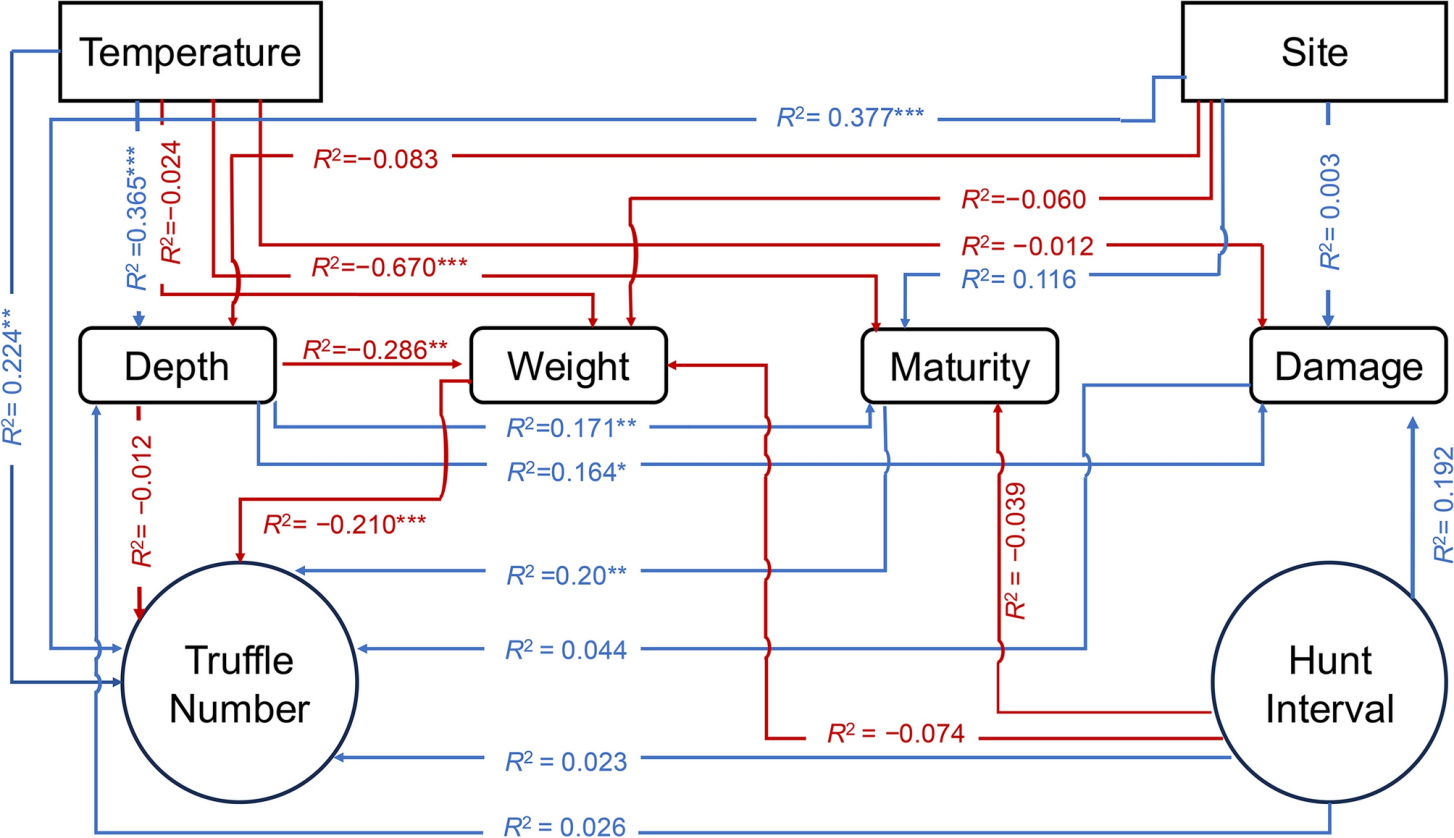
Partial least squares structural equation model (PLS-SEM) showing the causal relationships between location, temperature, depth of collection, truffle quality and hunt interval that influenced dog behaviour and truffle harvest. Circles and rectangles denote latent and indicator variables respectively. Red lines denote negative relationships while the blue lines represent positive interactions. Asterisks next to the path coefficient (*R*^2^) values indicate significance, *** *p* ≤ 0.0001; ** *p* ≤ 0.01; * *p* ≤ 0.05.

The PLS-SEM model (Fig. 8) constructed to evaluate causal relationships between location, temperature, depth of collection, truffle quality (weight, maturity and damage) and hunt interval revealed that location significantly impacted the numbers of truffle collected (*p* < 0.0001) but had no effect on the weight, maturity or damage to truffles (Fig. 8). While hunt location had no effect on the depth of collection, ambient temperatures of the hunt location significantly influenced the collection depth of the truffles (*p* < 0.0001). Temperature had a determining effect on the numbers of truffles collected (*p* = 0.0009) and maturity of truffles (*p* < 0.0001) but did not have significant effects on damage or weight of the truffles.

The interval between hunts did not affect depth of collection or numbers of truffles collected (Fig. 8). The depth of collection had a negative influence on truffle weight (*p* = 0.008) but positively affected truffle maturity (*p* = 0.002) and damage (*p* = 0.02). Depth of collection had no effect on the numbers collected. The length of interval between hunts influenced the maturity of truffles (*p* = 0.03), truffles matured more when duration between the hunts was longer. A positive relationship (*p* < 0.0001) was found between the maturity of the truffles and the numbers collected i.e. greater numbers of mature truffles were collected by the hunters.

## Discussion

### Truffle seasonality and eco-physiological interaction

The average weight of 31.1 g observed from a total of 3180 harvested truffles (Fig. 1), is startling close to the 33 g recorded in another large dataset reported by Büntgen et al*.*^7^,. Moreover, a dataset of 1003 collected truffles of the related species, *Tuber melanosporum*, displayed an average weight of 34 g with no difference between two tested irrigation regimes^19^. Despite our expectation that truffle size is influenced by environmental variables such as precipitation, the convergence of datasets across different countries and irrigation regimes suggests that this may not be the case. Indeed, we see here that average truffle weight over the harvesting season does not change (Supplementary Fig. 1B) despite significant seasonal shifts such as average temperatures of 18 °C in March to 33 °C in July (Fig. 2C; Fig. 8). This de-coupling of truffle weight with climate counters expectation and is interesting from an ecophysiology perspective, but also suggests that cultivators may not be able to use irrigation to alter truffle-weight within orchards to meet market expectations.

We also explored if truffle weight is related to maturity, i.e. are bigger truffles also more mature? Previous authors showed no correlation between weight and maturity for *T. aestivum*^7^ nor the related species *T. melanosporum*^20^ but within this dataset, we see that although all truffle weights can correspond to all maturity grades, there is a relationship. Lower truffle weights were most prevalent in the highest maturity categories and further, depth of collection positively correlates with maturity and negatively with weight. Here, we hypothesise that it is not weight and maturity that are entangled, but rather that fruiting/collection depth is the key driver of this relationship, and this is explored further in 4.2.

In terms of mycophagy, truffle damage was greater at higher maturity grades, and this is likely explained by the dependency of truffles on mycophagy for the distribution of colonies in time and space, as well as successful fruiting through mating-type interaction^4–6^. Truffles use aroma to attract predation and the concentration of many volatiles increase with maturation^21^ as do the levels of possible non-aromatic chemoattractants^22^. As such, higher levels of truffle damage are expected with increased maturation.

Finally, it is widely accepted by truffle hunters/cultivators that the maturity of collected truffles increases as the season progresses, and here we provide evidence that clearly validates this observation. However, although the size of recovered truffles doesn’t seem to show a seasonal pattern, the quantity of recovered truffle does. This latter observation presents as the season ‘warming-up’ in terms of numbers of fruit bodies recovered, before peaking and again slowly declining: this displays an expected progression in-line with previous studies^7,19^. Finally, despite the interaction of mycophagy and truffle maturity we don’t see a seasonal progression of truffle damage and it’s important to note that this is a proportional value and so the amount of mycophagy/damage is increasing in quantitative terms but the proportion of the harvest that is damaged remains the same. These are all important points for truffle hunters/cultivators and will help to inform behaviour ‘on the ground’.

### Fruiting depth correlates with truffle quality – dog ethology

Garcia-Barreda et al*.*^20^*,* using a total of seven hunt days per site, reported that fruiting depth does not correlate with truffle maturity. However, using a larger and broader dataset we report here, and for the first time, that fruiting/harvesting depth indeed has a significant causal influence on truffle quality (Fig. 8). Within this we see a conflict, deeper truffles were more mature (Fig. 3) and therefore of higher value, but they were also more likely to be subject to higher levels of mycophagy, a factor which reduces market acceptance^23^. First, we explore why these relationships exist. Čejka et al*.*^3^*,* showed that on consecutive day truffle hunting within the same area, the second day’s crop was found at a greater depth. From a dog perspective scent penetration is impacted by soil type^24^, but also soil depth^25^ and therefore, shallower truffles are likely easier for a truffle-dog to detect. Framed in this context, the relationship we see here between truffle maturity and depth of fruiting can be explained by deeper truffles being less easily detected and therefore harvested – the dogs quickly detect the ‘low hanging fruit’ of shallow truffles whilst deeper truffles require more focus and time. The deeper truffles may not be located as frequently as the dogs fail to invest enough time and effort to their detection, focussing on a quick and easy reward or it may be the handler losing patience with the longer time needed to detect truffles and moving the hunting team along. Whether the driver is the dog or handler, we suggest that those deeper truffles remain in the ground longer with a greater chance of reaching an advanced stage of maturity. This greater ‘soil persistence’ time also explains the higher degree of damage by mycophagy we observed in deeper forming truffles. These two measures of truffle quality are therefore a likely artefact not of ecophysiology but of truffle dog or handler ethology. Beyond some proposed hypotheses by Čejka et al*.*^3^, dog and handler ethology has never been associated with truffle quality metrics and here we present a theory that likely links the two. This raises the practical consideration that if a hunter wishes to harvest deeper truffles more frequently, perhaps to reduce the recovered proportion that are damaged or indeed to reduce the quantity of fruitbodies that remain undetected^8^, dog-training can be targeted accordingly. These insights may facilitate an informed approach by practitioners, which could be widely beneficial and further suggest that the percentage of truffles that are collected from deeper in the soil profile may be a good proxy for the efficacy of a hunt team. Moreover, these results highlight the significant and serious need to address dog ethology in studies that depend on truffle collection for data analysis. However, although dog ethology is a likely significant driver of the relationships we observe here, there are other explanations that may also contribute. As discussed by Thomas and Thomas^4^ invertebrates that predate truffles occur throughout the soil profile and may concentrate in deeper zones to escape desiccation in arid environments. The study area is within one of the dryer regions of Europe and this may be a driver of increased mycophagy at depth, although countering this is the fact that we see no proportional change in damage across the whole fruiting period of March − July whilst temperatures increase. It should also be noted that most of the recorded mycophagy damage will likely have been caused by invertebrates, as vertebrates such as mammals are more likely to remove the target truffle from a location, collect from higher in the soil profile, or occasionally consume in its entirety^6,26,27^.

Finally, even though we suggest that targeted dog training can help to recover deeper truffles before they are damaged and increase collection quantity, caution should be applied. For example, it may be the case that deeper truffles are more impactful from a spore distribution perspective and therefore increased retrieval may be detrimental to future harvests (see 4.1) although there are interventions to address this, such as adding spores in a targeted manner^28^.

### From a hunter perspective—hunt intervals and optimisation

We hypothesised that a longer hunt interval would result in a greater truffle harvest per hunt, and although we found that this was not the case (see Fig. 3) we do see that a longer hunt interval is correlated with greater truffle maturity. This latter point is explained by a longer un-disturbed maturation period for the truffle within the soil environment.

The relationship between hunt interval and site was strongly significant (Fig. 3). Here we see that the hunters intuitively dedicate more hunting days to the sites that are producing the highest quantity of truffles (see Supplementary Fig. 4) and thus display a good degree of hunt-optimisation in terms of spatial distribution of inputs. Finally, towards the end of the season we see that although truffles collected per hunt event are still high, they do begin to decline before the final collection which occurs with an abrupt end to the fruiting season (see methods). This holistic and detailed exploration across a broad spatiotemporal scale, allows us to present causal relationships of truffle eco-seasonal variation and the interaction of dog/hunter ethology (see Fig. 8).

## Conclusion

Here we report in a systematic manner that both truffle quantity and quality change throughout the harvesting season, and some metrics such as truffle weight and maturity can be linked. We also observe that damage through mycophagy is clearly related to truffle maturity and that there is an unexpected de-coupling of truffle weight with climate variables.

This is also the first study to interpret the impact of dog ethology on truffle hunt success metrics such as the quantity and quality of recovered truffle, alongside the behaviour of truffle hunters in hunt optimisation. These insights are invaluable for practitioners and further suggest that dog ethology is an essential consideration for truffle ecological studies that focus on harvested fruitbodies. Taken as a whole, the study presents a comprehensive overview of how different truffle metrics are interlinked and influenced by both location and time, with dog and hunter ethology influencing collected truffle quality and quantity. As emerging fields, the results highlight that truffle dog and hunter ethology merit further study and consideration.

## Supplementary Information

Supplementary Information.

## Data Availability

Data is provided within the manuscript or supplementary information files and further details are available from the corresponding author upon reasonable request.

## References

[1] Čejka, T. et al. Risk and reward of the global truffle sector under predicted climate change. *Environ. Res. Lett.* **17**, 024001 (2022).

[2] Reyna, S. & Garcia-Barreda, S. Black truffle cultivation: A global reality. *For. Syst.* **23**, 317–328 (2014).

[3] Čejka, T. et al. Understanding the performance of truffle dogs. *J. Vet. Behav.* **52–53**, 8–13 (2022).

[4] Thomas, P. W. & Thomas, H. W. Mycorrhizal fungi and invertebrates: Impacts on Tuber melanosporum ascospore dispersal and lifecycle by isopod mycophagy. *Food Webs.* **33**, e00260 (2022).

[5] Ori, F. et al. Effect of slug mycophagy on Tuber aestivum spores. *Fungal Biol.* **125**, 796–805 (2021). 34537175 10.1016/j.funbio.2021.05.002

[6] Urban, A. Truffles and small mammals. In *true truffle (Tuber spp.) in the world: Soil ecology, systematics and biochemistry* (ed. Alessandra Z., Mirco I., Claude M.,) 353-373 (Springer International Publishing, 2016)

[7] Büntgen, U. et al. New insights into the complex relationship between weight and maturity of burgundy truffles (Tuber aestivum). *PLOS ONE* **12**, e0170375 (2017). 28125633 10.1371/journal.pone.0170375 PMC5268403

[8] Schneider-Maunoury, L., Taschen, E., Richard, F. & Selosse, M. A. Soil spore bank in Tuber melanosporum: up to 42% of fruitbodies remain unremoved in managed truffle grounds. *Mycorrhiza* **29**, 663–668 (2019). 31701214 10.1007/s00572-019-00912-3

[9] Centenaro, G. et al. The importance of botanic gardens for global change research—New insights into Cambridge’s hidden truffle kingdom. *PLANTS, PEOPLE, PLANET* **5**, 329–334 (2023).

[10] Gezer, K., Kaygusuz, O., Çelik, A. & Işiloǧlu, M. Ecological characteristics of truffles growing in Denizli province. *Turkey. J. Food Agric. Environ.* **12**, 1104–1109 (2014).

[11] Alonso Ponce, R. et al. Soil physical properties influence “black truffle” fructification in plantations. *Mycorrhiza* **24**, 55–64 (2014). 24487451 10.1007/s00572-014-0558-7

[12] Todesco, F. et al. Soil temperature and hydric potential influences the monthly variations of soil Tuber aestivum DNA in a highly productive orchard. *Sci. Rep.*10.1038/s41598-019-49602-2 (2019). 31506577 10.1038/s41598-019-49602-2 PMC6736833

[13] R Core Team. R: a language and environment for statistical computing. R Foundation for Statistical Computing, Vienna, Austria. https://www.R-project.org (2023).

[14] Gross, J., & Ligges, U. nortest: tests for normality. R package version 1.0–4. https://CRAN.R-project.org/package=nortest (2015).

[15] Peterson, R. A. Finding optimal normalizing transformations via bestnormalize. *R J.* **13**, 310 (2021).

[16] Wei, T. & Simko, V. R package ‘corrplot’: visualization of a correlation matrix (Version 0.92). https://github.com/taiyun/corrplot (2021).

[17] Kassambara, A. ggcorrplot: visualization of a correlation matrix using ‘ggplot2’. R package version 0.1.4.1. https://CRAN.R-project.org/package=ggcorrplot (2023).

[18] Ray, S., Danks, N. & Valdez, A. C. seminr: building and estimating structural equation models. R package version 2.3.2. https://cran.r-project.org/package=seminr (2022).

[19] Magarzo, A. et al. Effect of irrigation methods on black truffle production. *Agronomy* **13**, 2505 (2023).

[20] Garcia-Barreda, S., Sánchez, S., Marco, P., Benucci, G. M. N. & González, V. Lack of linkages among fruiting depth, weight, and maturity in irrigated truffle fungi marks the complexity of relationships among morphogenetic stages. *J. Fungi* **7**, 102 (2021). 10.3390/jof7020102 PMC7912816 33535599

[21] Caboni, P. et al. Multi-platform metabolomic approach to discriminate ripening markers of black truffles (Tuber melanosporum). *Food Chem.* **319**, 126573 (2020). 32169760 10.1016/j.foodchem.2020.126573

[22] Pacioni, G. et al. Truffles contain endocannabinoid metabolic enzymes and anandamide. *Phytochemistry* **110**, 104–110 (2015). 25433633 10.1016/j.phytochem.2014.11.012

[23] Rosa-Gruszecka, A. et al. Insect-truffle interactions – potential threats to emerging industries?. *Fungal Ecol.* **25**, 59–63 (2017).

[24] Alexander, M. B., Hodges, T. K., Wescott, D. J. & Aitkenhead-Peterson, J. A. The effects of soil texture on the ability of human remains detection dogs to detect buried human remains. *J. Forensic Sci.* **61**, 649–655 (2016). 27122400 10.1111/1556-4029.13084

[25] Dargan, R. & Forbes, S. L. Cadaver-detection dogs: A review of their capabilities and the volatile organic compound profile of their associated training aids. *WIREs Forensic Sci.*10.1002/wfs2.1409 (2020).

[26] Fogel, R. & Trappe, J. M. Fungus consumption (mycophagy) by small animals. *Northwest Sci.* **52**, 1–31 (1978).

[27] Elliott, T. F. et al. Mammalian mycophagy: A global review of ecosystem interactions between mammals and fungi. *Fungal Syst. Evol.* **9**, 99–159 (2022). 36072820 10.3114/fuse.2022.09.07 PMC9402283

[28] Taschen, E. et al. Efficiency of the traditional practice of traps to stimulate black truffle production, and its ecological mechanisms. *Sci. Rep.*10.1038/s41598-022-19962-3 (2022). 36171390 10.1038/s41598-022-19962-3 PMC9519532

