# Supplementary material for: Hunting dog behaviour is a key driver impacting harvest quantity and quality of truffles
Source: Sci Rep. 2025 Mar 13;15:8662. doi: 10.1038/s41598-025-93116-z (PMC11906787; doi:10.1038/s41598-025-93116-z)

Supplementary information. Truffle ecophysiology, hunting dog behaviour and dog handler bias influence harvest quality and quantity. Thomas, P.W., Kothamasi, D.

Supplementary Figure 1. Effects of (A) collection site and (B) month of collection on *T. aestivum* truffe weight. Values that do not share an alphabet are significantly different ($p \leq 0.05$) in a Tukey's post-hoc test for pairwise differences. The centre line in the box and whisker plots indicate the median and the blue dots denote the mean. The upper and lower whiskers indicate the highest and lowest values while circles above whiskers are the outliers.

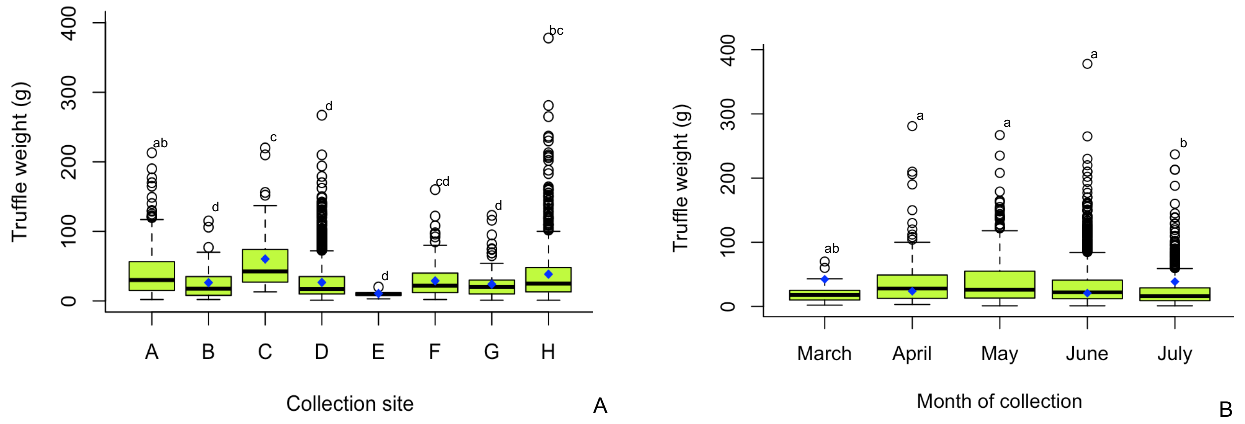

Supplementary Figure 2. Effect of maturity grade of *T. aestivum* truffle on its (A) weight and (B) damage grade. Values that do not share an alphabet are significantly different ($p \leq 0.05$) in a Tukey's post-hoc test for pairwise differences. The centre line in the box and whisker plot indicates the median and the blue dots denote the mean. The upper and lower whiskers indicate the highest and lowest values while circles above whiskers are the outliers. Error bars in the bar chart represent standard error of the mean.

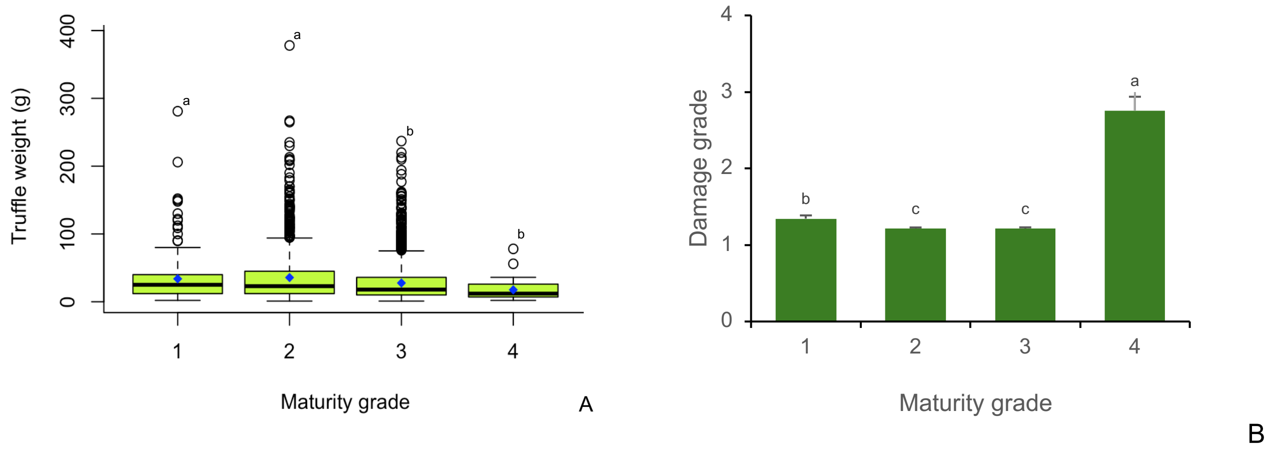

Supplementary Figure 3. Correlation matrix showing the interrelationships between site, truffle quality characteristics, depth of collection and temperature. Correlations were computed based on parameters measured for 3180 truffles of *T. aestivum* collected from eight sites over five-month harvesting season

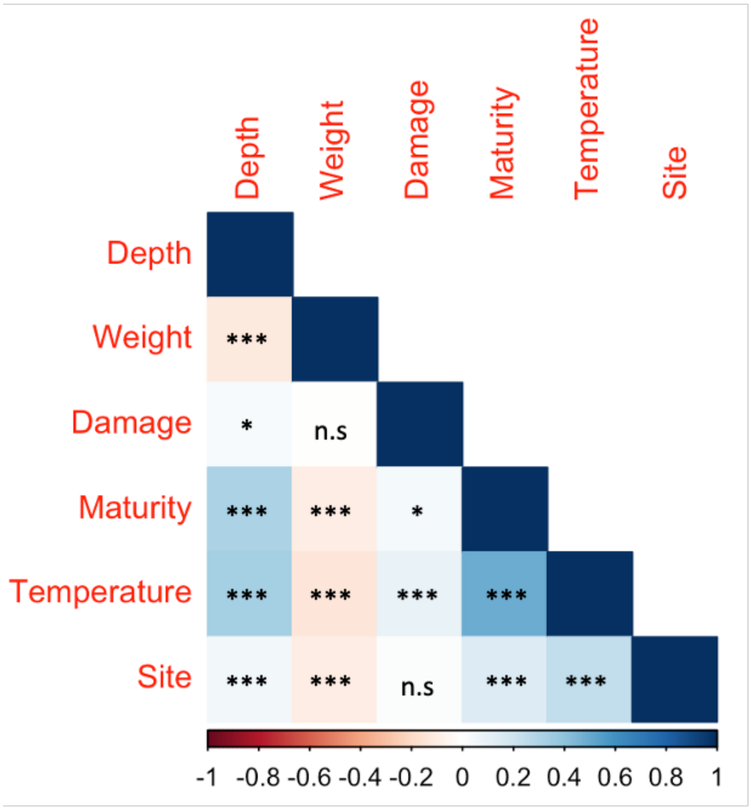

Correlation coefficient *r*

*** < 0.001; ** < 0.01; * < 0.05; n.s: Not significant

Supplementary Figure 4. Duration of interval between truffle hunts at each collection site. Hunt intervals were shortest in sites with highest truffle yields.

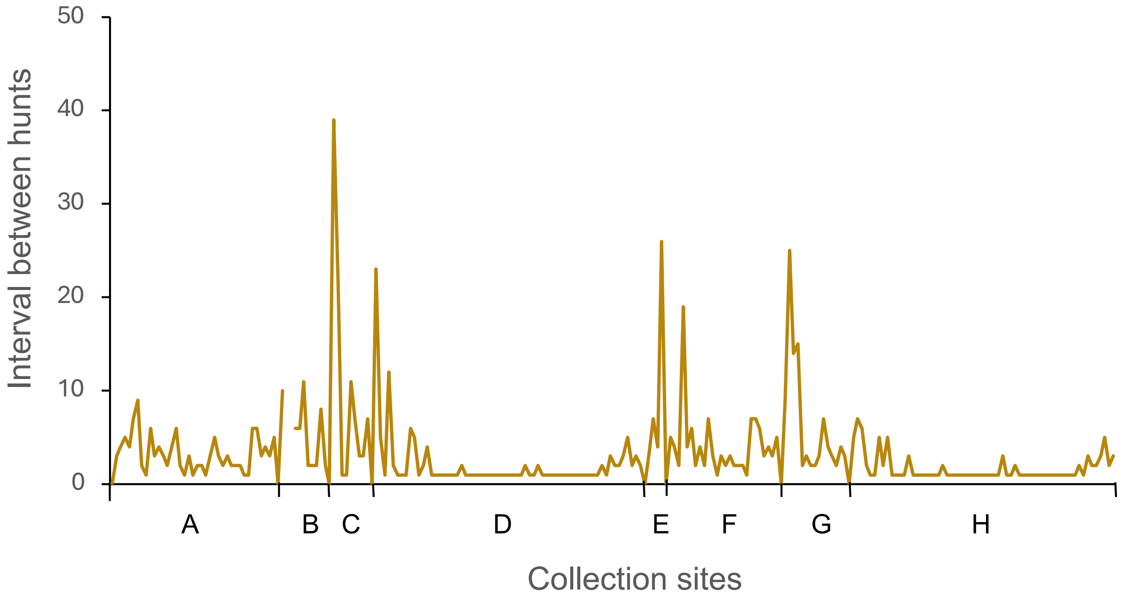

Supplement: Supplementary file 1 — Supplementary Information. [file 41598_2025_93116_MOESM1_ESM.pdf]
